# Galectins in the Pathogenesis of Rheumatoid Arthritis

**DOI:** 10.4172/2155-9899.1000164

**Published:** 2013-09-30

**Authors:** Song Li, Yangsheng Yu, Christopher D Koehn, Zhixin Zhang, Kaihong Su

**Affiliations:** 1 Department of Pathology and Microbiology, University of Nebraska Medical Center, Omaha, NE 68198, USA; 2 Department of Internal Medicine, University of Nebraska Medical Center, Omaha, NE 68198, USA; 3 The Eppley Cancer Institute, University of Nebraska Medical Center, Omaha, NE 68198, USA

**Keywords:** Rheumatoid arthritis, Galectin, Inflammation, Pathogenesis, T cells, Fibroblast-like synoviocytes

## Abstract

Rheumatoid arthritis (RA) is a complex and common systemic autoimmune disease characterized by synovial inflammation and hyperplasia. Multiple proteins, cells, and pathways have been identified to contribute to the pathogenesis of RA. Galectins are a group of lectins that bind to β-galactoside carbohydrates on the cell surface and in the extracellular matrix. They are expressed in a wide variety of tissues and organs with the highest expression in the immune system. Galectins are potent immune regulators and modulate a range of pathological processes, such as inflammation, autoimmunity, and cancer. Accumulated evidence shows that several family members of galectins play positive or negative roles in the disease development of RA, through their effects on T and B lymphocytes, myeloid lineage cells, and fibroblast-like synoviocytes. In this review, we will summarize the function of different galectins in immune modulation and their distinct roles in RA pathogenesis.

## Introduction

Rheumatoid arthritis (RA) is a complex and common systemic autoimmune disease, characterized by synovial inflammation and hyperplasia, cartilage and bone destruction, and extra-synovial symptoms [1]. The prevalence of RA in the adult population is estimated at 1% worldwide and is three times higher in women than in men [2]. RA principally attacks flexible joints symmetrically, progressing from distal joints to proximal joints [3]. RA inflammation can also diffuse into extra-synovial tissues and organs, leading to a higher risk of developing cardiovascular diseases, lymphoma, and lung cancer [2,4,5]. The diagnosis of clinical RA is based on several criteria, including physical symptoms, joint radiographs, and serological tests [6]. Treatment strategies for RA patients include non-steroidal anti-inflammatory drugs (NSAIDs), disease-modifying anti-rheumatic drugs (DMARDs), and biological agents, such as blocking antibodies for tumor necrosis factor alpha (TNFα) and interleukin-6 (IL-6) [2]. Although these drugs can relieve symptoms and delay disease progression, none of them provide a cure for RA nor have consistent efficacy in all patients.

The etiology of RA involves a complex interplay of multiple proteins, cells, and pathways. Among those, galectins have recently emerged as an important group of proteins which modulate immune activation and inflammation [7]. Galectins are the lectin family members that bind to β-galactoside carbohydrates. They are widely expressed in different tissues and organs with the highest expression patterns in the immune system [8]. Through binding to their receptors, galectins mediate fundamental intra- and inter-cellular signaling as well as cell-extracellular matrix (ECM) interactions [8]. As potent immune regulators, galectins play an important role in a number of pathological processes including inflammation, autoimmunity, fibrosis, and cancer [7].

In this review, we will summarize the current understanding of the role of different galectins in RA, based on a comprehensive literature review of published empirical research. The electronic databases of Pubmed/Medline, Embase, EBSCO, SCOPUS, and Cochrane Library were searched using key words “arthritis” and “galectin” in all fields to the cut-off date of September 16, 2013. Over 100 manuscripts in English language were identified in the search. Among those, thirty research manuscripts and one conference abstract provide direct evidence regarding the pathogenic role and therapeutic potential of galectins in RA (Table 1). Herein, we will briefly review the pathogenic mechanisms of RA and discuss in detail the role of different galectins in RA pathogenesis and therapeutics.

## Pathogenesis of RA

Although RA was first described more than 200 years ago, its etiology has not been completely characterized. Both genetic and environmental factors contribute to the development of RA. To date, more than 30 gene loci have been found to contribute to RA susceptibility and disease severity [9–11]. Many of those gene loci are related to immune cell activation, such as MHC class I allele HLA-DRB1 and gene variants of cytotoxic T lymphocyte-associated antigen-4 (CTLA-4) and proteintyrosine-phosphatase nonreceptor type 22 (PTPN22). Environmental risk factors include bacterial and viral infections, smoking, and alcohol consumption [11,12]. Gene-environment interactions can also synergistically increase the risk of developing RA in certain subgroups of people. For example, a combination of smoking and the HLA-DRB1 allele increases the risk for RA by 21-fold in the anti-cyclic citrullinated peptide antibody (ACPA) positive population [13].

A major characteristic of RA is the infiltration of multiple leukocytes into the joints, including B cells, T cells, macrophages, dendritic cells, and neutrophils. Infiltrated leukocytes form ectopic germinal centers and drive adaptive immune responses in the RA joints. B cells can locally produce autoantibodies, including ACPA [14,15]. T cells play a central role in mediating joint damage by driving the activation of other effector cells [16,17]. Although CD4^+^ T cells are the dominant T cell types in the synovium, Th17, a subset of T helper cells secreting IL-17, and regulatory T cells (Treg) also play a critical role in RA pathogenesis [18,19]. Neutrophils are the most abundant leukocytes in RA synovial fluid (SF) [20]. In RA patients, SF neutrophils remain active for an overly extended length of time [21,22]. Activated neutrophils release proteolytic enzymes, reactive oxygen species (ROS), and neutrophil extracellular trap (NET), which can damage local tissues as in other autoimmune diseases [23]. Neutrophils also secrete pro-inflammatory cytokines such as TNFα, IL-1 and IL-6, as well as chemokines to further amplify joint inflammation [24]. Macrophages, derived from circulating monocytes or local macrophage-like synoviocytes, provide another main source of pro-inflammatory cytokines [25]. In addition, synovial macrophages in RA strongly express MHC class II and are potent for antigen presenting and T cell activation [16].

Another characteristic of RA is the activation of local fibroblast-like synoviocytes (FLS) [26]. In RA joints, resident FLS show a transformed phenotype with over-expressed proto-oncogenes and defective cell death pathways. FLS in RA secret a wide range of pro-inflammatory cytokines and chemotactic proteins and also express surface ligands for interacting with immune cells [27]. In addition, RA FLS release proteolytic enzymes such as matrix metalloproteinases (MMP), cathepsins, and plasmins. Thus, it has been suggested that FLS are the main cells responsible for the invasion and destruction of cartilage and bones, promotion of angiogenesis, and facilitation of osteoclastogenesis [27].

Besides the cells mentioned above, numerous proteins have been shown to play a role in RA pathogenesis. Some of them have been successfully adapted in clinical diagnosis and therapies for RA, such as ACPA, TNFα, IL-1, and IL-6 [2]. The family of galectins is involved in a wide range of biological processes. Their immune modulating role has drawn an increasing attention in the field of arthritis research. Our discussion will now turn to the function of galectins and their potential role in RA pathogenesis and therapies.

## The Family of Galectins

**Figure 1:**
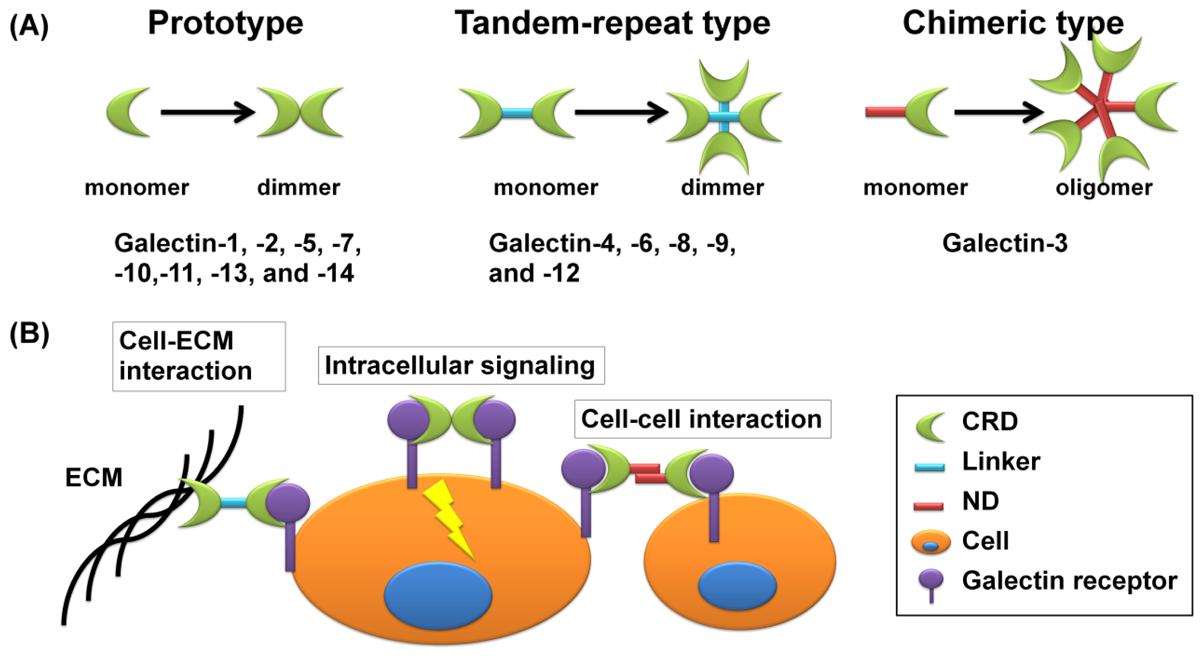
The structure and function of the galectin family members **(A)** The galectin family members are divided into three types: the prototype with one carbohydrate recognition domain (CRD), the tandem-repeat type with two CRDs connected by a non-conserved linker, and the chimeric type with one CRD and a non-lectin N-terminal domain (ND). Some galectins can self-associate into dimers or oligomers. **(B)** Biological functions of extracellular galectins. Bivalent or multivalent galectins crosslink their receptors on the same cell for intracellular signal transduction, two different cells for cell-cell interaction, or cell and extracellular matrix (ECM) for cell-ECM interaction.

Galectins are a group of lectins that specifically bind to β-galectoside carbohydrates and share significant sequence similarity in their carbohydrate-recognition domains (CRDs) [8]. The galectin genes are evolutionarily conserved and can be found in many organisms, including viruses, sponges, fungi, plants, nematodes, insects, and vertebrates [8]. Currently there are at least 15 mammalian galectins, all of which contain one or two CRDs of about 130 amino acids each. Based on the CRD organization, galectins are divided into three subfamilies (Figure 1, panel A). Galectin-1, -2, -5, -7, -10, -11, -13, and -14 contain only one CRD and are classified as the “proto type”. In contrast, the “tandem-repeat type” (galectin-4, -6, -8, -9, and -12) have two separate CRDs connected by non-conserved amino acid sequences. Galectin-3 is the only member of the “chimeric type” and contains one CRD and a non-lectin region of about 120 residues at the N-terminal of CRD [8]. Some galectins can self-dimerize or oligomerize, forming bivalent or multivalent complexes for stronger signaling [28,29] (Figure 1, panel A).

Galectins have been detected in numerous tissues and organs. Their distributions can be ubiquitous (*e.g*. galectin-1, -3, -8 and -9) or limited to specific tissue types (*e.g*. galectin-2 and -4) [30]. Due to the absence of the classical signal sequence for insertion into the endoplasmic reticulum (ER), galectins primarily localize intracellularly [31]. However, some types of galectins can be found on the cell surface (e.g. galectin-9) or secreted through a non-classical ER/Golgi-independent pathway to the extracellular compartment (e.g. galectin-1 and -3) [32,33].

## Function of Galectins

The sugar-binding specificity and affinity vary among different members of the galectin family, implicating their specialized and diversified functions [34]. Each galectin recognizes a set of glycoproteins with a particular oligosaccharide sequence. The variety of binding partners and wide distribution of galectins allow them to function in multiple biological reactions, including mRNA splicing (e.g. galectin-1 and -3) [35,36], cell apoptosis (e.g. galectin-3, 7,-9,-12) [37–39], cell cycle regulation (e.g. galectin-3 and -12) [40,41], cell activation (e.g. galectin-3) [42,43], cell adhesion and migration (e.g. galectin-1, -2, -3, -4, -8 and -9) [44], and cell differentiation (e.g. galectin-3, -9, -10) [45]. Pathologically, galectins have been linked to a number of diseases including cancer, cardiovascular disease, liver fibrogenesis, asthma, and RA [7].

The role of galectins in RA varies among different members of the galectin family as different galectins can positively or negatively regulate immune responses and inflammatory reactions. To date, multiple studies have identified a regulatory role of galectin-1, -3, and -9 in RA while only a few studies suggested a role of galectin-2 and -8 in RA. We will now discuss each of the five types of galectins regarding to their potential function in RA.

## Galectin-1 in RA

### Overview of galectin-1

Galectin-1 is a “proto type” galectin and can form homodimers by cross-linking [46,47]. It is highly expressed by immune-related cells such as lymphoid stromal cells, macrophages [48], T cells [49], and endothelial cells [50]. In most studies, galectin-1 has been shown to be immunosuppressive and anti-inflammatory. The main receptors of galectin-1 on the T cell surface are CD43 and CD45 [51,52]. Through surface receptor binding, galectin-1 regulates negative selection of T cells in the thymus [51,53], induces Th1 and Th17 cell apoptosis [54], and promotes the shift from Th1 to Th2 polarized immune responses [55]. Treatment of T cells with galectin-1 changes the cytokine profile, with decreased pro-inflammatory cytokines such as TNFα, IL-1β, IL-2, and IFNγ [56,57] and increased anti-inflammatory cytokines such as IL-10 [58]. For B cells, galectin-1 negatively regulates cell proliferation and BCR-mediated signal transduction [59]. Galectin-1 also regulates innate immune cell activation. Treatment of galectin-1 dramatically reduced neutrophil infiltration, mast cell degranulation [60], and inducible nitric oxide synthase (iNOS) expression in macrophages [61]. The anti-inflammatory activity of galectin-1 has also been suggested in various experimental models of inflammatory or autoimmune diseases including experimental autoimmune uveitis [62], myasthenia gravis [63], graft-versus-host disease [64], experimental autoimmune encephalomyelitis [65], experimental colitis [66], diabetes [67], concanavalin A-induced hepatitis [68], and collagen-induced arthritis [55].

### Galectin-1 in arthritis animal models

The link between galectin-1 and RA was first reported by Rabinovich et al. in 1999 using collagen-induced arthritis (CIA) mouse model [55]. A single injection of fibroblasts engineered to secrete mouse galectin-1 or daily administration of 100 μg of recombinant human galectin-1 in DBA/1 mice was sufficient to suppress the overall clinical and histopathological manifestations of CIA [55]. Galectin-1 treatment also reduced the anti-collagen antibody levels and skewed the cytokine profile toward a type-2 polarized immune reaction [55]. Further investigation into the mechanism revealed that galectin-1 treatment enhanced the susceptibility of T cells to antigen-induced apoptosis, increased T cell adhesion to extracellular matrix, and also inhibited IL-2 secretion from collagen-specific T cell hybridomas [54,56,57]. In addition, galectin-1 functions to limit neutrophil recruitment to TNF-treated endothelium; and leukocyte adhesion and emigration were significantly increased in galectin-1-deficient mice inflamed with IL-1β [69]. In a more recent study, galectin-1-deficient mice exhibited increased susceptibility to CIA, with earlier onset of arthritis and more severe manifestations than the wild type mice [70]. These studies further demonstrated the inhibitory function of galectin-1 in the development of arthritis and the disease severity in animal models.

### Galectin-1 in RA patients

In human patients, *in situ* immunohistochemistry showed remarkably reduced expression of galectin-1 in synovial tissue from patients with long duration of juvenile idiopathic arthritis (JIA) [71]. Reduced expression of galectin-1 may lead to defective mononuclear cell apoptosis in JIA patients [71]. Furthermore, expression of galectin-1 has never been found at the sites of cartilage invasion in RA [72,73]. Although the plasma levels of galectin-1 are comparable between RA patients and healthy controls, concentration of galectin-1 in synovial fluid (SF) is significantly decreased [74]. The reduced SF galectin-1 levels correlate to the increased levels of anti-galectin-1 autoantibodies and anti-cyclic citrullinated peptide (CCP) antibodies in RA patients [74]. These clinical studies confirmed the potential involvement of galectin-1 in RA pathogenesis, and provided a rational for using synovial galectin-1 as a biomarker for RA prognosis.

### Therapeutic potential of galectin-1

Based on the immunomodulatory effects of galectin-1 in RA, there has been much interest in designing galectin-1 derivatives as anti-RA drugs. In one study, intra-articular lentiviral vectors encoding galectin-1 were injected into rats with CIA [75]. This treatment significantly ameliorated CIA, measured by articular index, radiographic, and histological scores; T-cell infiltrates; and microvessel density in the ankle joints [75]. High frequencies of antigen-induced T cell apoptosis were also noticed in the lymph nodes of treated rats [75]. However, the anti-RA activities of galectin-1 require concentrations higher than 7 μM to allow formation of galectin-1 homodimers [76,77]. To overcome this limitation, galectin-1 was conjugated onto gold nanoparticles (Au-Gal1) to form a multivalent structure [78]. Au-Gal1 provided enhanced stability and biological activity, and showed better therapeutic effects than free galectin-1 *in vitro* and *in vivo*. In another study, a chimeric protein was genetically engineered by fusing galectin-1 to the Fc region of human IgG1 (Gal-1hFc) [79]. Gal-1hFc is stable and always dimeric, thus the molecule is biologically functional at low concentrations. Investigation of Gal-1hFc’s effects on leukocytic infiltrates in RA synovial fluids showed that 94% of leukocytes expressed galectin-1 receptor and were susceptible to Gal-1hFc–mediated cell death, revealing the potency of this chimeric protein for RA treatment. Furthermore, a recent study showed that low concentrations of galectin-1 can induce chondrogenic differentiation of mesenchymal stem cells (MSCs) from RA bone marrow [80], suggesting a potential application of galectin-1 in cartilage transplantation treatment for RA.

In summary, galectin-1 plays an inhibitory role in the development of experimental arthritis mainly through the induction of T cell apoptosis and skewed type-2 cytokine response. In human patients, the expression levels of galectin-1 were significantly down-regulated in the synovium of RA and JIA patients and the downregulation of galectin-1 was correlated with the increased anti-CCP titers. In pre-clinical animal studies, administration of galectin-1 or its derivatives ameliorated the antigen-induced arthritis, providing a strong rational for using galectin-1 as anti-RA drugs in the future.

## Galectin-3 in RA

### Overview of galectin-3

While galectin-1 is a negative regulator of autoimmunity in RA, galectin-3 promotes inflammation in RA. Galectin-3 is the only chimeric type of galectin. It has a long N-terminal domain with proline-and glycine-rich repeats connected to one CRD [81]. The N-terminal domain, which is 34% homologous to the collagen-1 chain, is responsible for self-oligomerization, and thus is essential for its biological activity [81]. Galectin-3 exists as monomer in solution, and self-assembles into higher order oligomers in the presence of multivalent carbohydrate ligands [82].

Functionally, galectin-3 is also known as epsilon BP for its IgE-binding activity and as Mac-2, a macrophage surface antigen [81]. By cross-linking cell surface receptors, galectin-3 activates several types of lymphoid and myeloid cells. It increases IL-2 production in T cells [83] and promotes IgE production in B cells [84]. For myeloid-linage cells, galectin-3 stimulates superoxide release from neutrophils and monocytes [43], potentiates IL-1 production by monocytes [85], and induces 5-hydroxytryptamine (5-HT) release from mast cells [86] and basophils [86,87]. In addition, galectin-3 can bridge cells and the ECM to promote chemo-attraction and retention of macrophages [88] and neutrophils [89]. In line with its *in vitro* pro-inflammatory function, it has been shown that the levels of galectin-3 are elevated in the serum or nidi of patients with inflammatory diseases including RA [72], systemic lupus erythematosus (SLE) [90], Behçet’s disease [91], and systemic sclerosis [92].

### Galectin-3 in arthritis animal models

Studies with CIA rats found increased galectin-3 secretion into the plasma over time, which correlated with the disease progression, implicating that galectin-3 promotes the development of experimental arthritis [93]. Recent studies with galectin-3-deficient mice further confirmed the stimulating role of galectin-3 in arthritis [94]. The joint inflammation and bone erosion of antigen-induced arthritis were markedly suppressed in galectin-3-deficient mice as compared with the wild type mice [94]. The reduced arthritis in galectin-3-deficient mice was accompanied by decreased levels of antigen-specific IgG and proinflammatory cytokines including TNFα, IL-6, and IL-17 [94]. Furthermore, an exogenous supply of recombinant galectin-3 restored the reduced arthritis and cytokine production in galectin-3-deficient mice [94]. This study provided the direct evidence that galectin-3 plays a crucial role in the development of arthritis in animal models.

### Galectin-3 in RA patients

In human patients, galectin-3 was detected in the synovial tissue of RA and JIA patients, with clear accumulation at the sites of cartilage invasion [71,72,95–97]. The serum levels of galectin-3 were elevated in patients with RA, JIA, Behçet’s disease, or systemic sclerosis [72,91,92,98]. Although the increased galectin-3 is not specific for RA, the serum levels of galectin-3 were significantly associated with the C-reactive protein (CRP) levels and the disease activity scores in patients with JIA, suggesting that galectin-3 may be utilized as a biomarker for the disease progression of JIA [98]. In addition, the galectin-3 gene allele (LGALS3 +292C) is more prevalent in RA patients than in healthy controls, indicating that genetic polymorphisms of galectin-3 may influence the susceptibility to RA [99].

In addition to immune cells, FLS in the synovium of RA patients also express galectin-3 at high levels [72,73,95]. While floating FLS only express low levels of galectin-3 [95], adhesion of FLS to cartilage components through CD51/CD61 induces galectin-3 expression [100]. In RA patients, about 39% of FLS are cartilage-adhering cells, which is four times more than in osteoarthritis (OA) patients. The increased numbers of adhering FLS contribute to the elevated galectin-3 levels in the RA synovium [100]. Moreover, galectin-3 can induce rheumatoid FLS to secret a set of pro-inflammatory cytokines and chemokines including IL-6, granulocyte-macrophage colony-stimulating factor (GMCSF), TNF, CXCL8, CCL2, CCL3, and CCL5 [101]. The induction of cytokines and chemokines by galectin-3 appears to involve different signaling pathways. The MAPK-ERK pathway was necessary for cyotokine IL-6 production, while phosphatidylinositol 3-kinase (PI3K) was required for chemokine CCL5 induction [101]. These studies using human materials further suggest a promotional role of galectin-3 in the pathogenesis of RA.

### Therapeutic potential of galectin-3

In concordance with the human and animal studies discussed above, silencing of galectin-3 expression by intra-articular injections of shRNA into rat ankle joints ameliorated the manifestation of CIA, suggesting that downregulation of galectin-3 may be a therapeutic strategy for RA [75]. In addition, using FLS derived from the synovium of RA patients, it has been reported that downregulation of galectin-3 expression by galectin-3 siRNA inhibited spontaneous and LPS-induced secretion of inflammatory cytokine IL-6, further suggesting the potential of targeting galectin-3 in the suppression of joint inflammation [102].

Overall, galectin-3 acts as a positive regulator for inflammation by stimulating proinflammatory cytokine/chemokine production and potentiating myeloid linage cell activation. In animal models, galectin-3 aggravates antigen-induced arthritis. In patients with RA and JIA, the levels of galectin-3 are increased in both serum and synovium. Thus, galectin-3 blockade may provide a novel strategy for the treatment of RA.

## Galectin-9 in RA

### Overview of galetin-9

Like galectin-1, galectin-9 is anti-inflammatory, as suggested by studies in several disease animal models including CIA [103,104], asthma [105], nephrotoxic serum nephritis [106], diabetic nephropathy [107], and autoimmune encephalitis [108]. Galectin-9 contains two distinct CRDs connected by a linker peptide [109]. Three isoforms of galectin-9 have been reported which differ in the length of the linker peptide: short type (311 AAs), medium type (323 AAs), and long type (355 AAs). Galectin-9 can also form stable dimers or multimers to induce stronger signals [110]. Galectin-9 is expressed by T cells, macrophages, endothelial cells, and fibroblasts and plays an important role in regulating inflammation and immune responses [111–113].

Galectin-9 negatively regulates pro-inflammatory T cell responses. An important cell surface receptor for galectin-9 is T cell immunoglobulin and mucin-domain-containing-molecule-3 (Tim-3). Tim-3 is specifically expressed on CD4^+^ Th1 cells, CD8^+^ cytotoxic T cells, and CD11b^+^ dendritic cells (DC), but not on Th2 cells or macrophages [114–116]. The galectin-9-Tim-3 pathway induces apoptosis of CD4^+^ Th1 and CD8^+^ cytotoxic T cells. Blockade of this interaction *in vivo* results in exacerbated autoimmunity and abrogation of self-tolerance in animal models [117]. Galectin-9 also regulates T cell subset differentiation *in vitro* and *in vivo*. In cell culture, treatment with galectin-9 induced the differentiation of naïve T cells to regulatory T cells (Treg) and suppressed the differentiation of Th17 cells [103].

### Galectin-9 in arthritis animal models

In mouse models, galectin-9 deficiency led to increased numbers of Th1 and Th17 cells and decreased numbers of Treg cells in the joint, rendering susceptibility to CIA [103]. Conversely, subcutaneous and intraperitoneal delivery of the human stable galectin-9 recombinant proteins decreased the production of proinflammatory cytokine and suppressed the disease symptoms in the CIA mice [103]. Another study by the same group demonstrated that treatment with human stable galectin-9 induced apoptosis of cells in the joints of CIA mice and SCID mice implanted with RA patient synovial tissues [103,104]. Furthermore, galectin-9 was shown to negatively regulate macrophage activation by increasing the expression of immunoinhibitory FcRIIb and decreasing the expression of immunoactivating FcRIII, leading to the suppression of arthritis in an immune complex-induced arthritis mouse model [118].

### Galectin-9 in RA patients

Using a cell culture system, stable galectin-9 protein preferentially induced apoptosis and suppressed the proliferation of RA patient-derived FLS [103,104]. In RA patients, decreased galectin-9-Tim-3 signaling has been observed. The levels of Tim-3 expression on CD4^+^ T cells from RA patients were lower compared to those from healthy controls, leading to blunted galectin-9-mediated apoptosis of CD4^+^ T cells [119,120]. Another study showed that galectin-9 mRNA expression levels in peripheral blood mononuclear cells (PBMCs) were significantly lower in RA patients with moderate to high disease activity than those with low disease activity [104,119], implicating that galectin-9 may prevent the disease progression of RA.

### Therapeutic potential of galectin-9

As discussed in the section of galectin-9 in arthritis animal models, administration of human stable recombinant galectin-9 ameliorated arthritis in CIA and an immune complex-induced arthritis mouse model, assessed by pannus formation, inflammatory cell infiltration, and bone/cartilage destruction [103,104]. These studies warrant the development of galectin-9 derivatives with enhanced *in vivo* stability and efficacy for the treatment of RA.

Taken together, galectin-9 is a negative regulator of arthritis as suggested by both animal and human studies. Galectin-9 plays a key role in T cell differentiation through the galectin-9-Tim-3 pathway. Galectin-9 induces the differentiation of naïve T cells to Treg cells and suppresses the differentiation of proinflammatory Th17 cells. In addition, galectin-9 induces apoptosis of FLS which may prevent synoviocyte hyperproliferation in RA joints. Therefore, up-regulation of galectin-9 and galectin-9-Tim-3 pathway is a promising strategy for the treatment of RA.

While galectin-1, 3, and 9 has been extensively studied regarding their modulating role in inflammation and arthritis, galectin-2 and -8 have been less studied in these aspects. Only a few reports revealed the linkage of galectin -2 and -8 with RA. In the following, we will briefly summarize these findings.

## Galectin-2 in RA

Galectin-2 is structurally similar to galectin-1, but has a distinct expression profile which is primarily confined to the gastrointestinal tract [121]. Like galectin-1, galectin-2 induces T cell apoptosis and suppressed colitis in a mouse model [122]. A human genetic study showed that galectin-2 3279C/T gene polymorphism may be independently associated with diastolic blood pressure in patients with RA [123]. These studies indicate that galectin-2 may play a suppressive role in RA, but more confirmative evidence is needed to support this notion.

## Galectin-8 in RA

Galectin-8 is a modulator of cell adhesion and cell growth [124]. The soluble form of galectin-8 was detected in the synovium of RA patients at the concentration that can induce apoptosis of synoviocytes [125]. However, the galectin-8-mediated apoptosis of synoviocytes was neutralized by free CD44vRA, a CD44 variant prevalent in RA SF [125]. In another study, function-blocking autoantibodies against galectin-8 were detected in a small percentage (about 20%) of RA patients [126,127]. The blockade of galectin-8 function in RA patients suggests that galectin-8 may play a suppressive role in RA. The potential role of galectin-8 in RA was further supported by a human association study. A single nucleotide polymorphism of galectin-8 that substitutes tyrosine for phenylalanine at position 18 was shown to be more prevalent in RA patients; and also associate with the early onset of RA in a large cohort [128].

## Conclusion

RA is a systemic autoimmune disease that involves a complex interplay of a variety of immune-modulating proteins [1]. Increasing amounts of evidence suggest that individual galectins, including galectin-1,-2, -3, -8, and -9, could play positive or negative roles in the pathogenesis of RA. Manipulation of certain types of galectins can suppress or aggravate the disease symptoms in arthritis animal models, indicating the therapeutic potential of galectins for the treatment of RA. Several anti-galectin compounds are under phase 1 or 2 clinical trials for the treatment of fibrosis and cancer. Although there are no ongoing clinical trials targeting galectins for the treatment of RA, we hope that galectin-related anti-inflammatory therapies will be developed in the future as we continue to unravel the specific immunomodulatory functions of individual galectins.

## Figures and Tables

**Table 1:** Pathogenic role and therapeutic potential of galectins in RA.

Galectin	Animal studies	Human studies	Therapeutic potential
**Galectin-1**	Administration of galectin-1 suppressed CIA by enhancing T cell apoptosis and inhibiting IL-2 secretion [55]. Galectin-1 limited neutrophil recruitment to inflammatory tissue by *in vitro* experiment and galectin-1-deficient mice [69]. Galectin-1 deficient mice were more susceptible to CIA [70].	Down-regulated expression of galectin-1 in the synovial fluid from RA/JIA patients [71,73,74].	Administration of galectin-1 or its derivates ameliorated CIA [55,75,78,79]. Galectin-1 induced chondrogenic differentiation of MSCs from RA bone marrow [80].
**Galectin-3**	Over-expression of galectin-3 was detected in CIA [93]. Galectin-3 deficient mice displayed reduced disease severity of antigen-induced arthritis [94].	Increased expression of galectin-3 in sera and synovial fluid in RA/JIA patients [71,72,91,95–97]. A galectin-3 gene allele (LGALS3 +292C) is more prevalent in RA patients [99]. RA patients had a higher number of galectin-3- expressing FLS [100]. Galectin-3 induced FLS to secret a set of proinflammatory cytokines and chemokines [101].	Intra-articular lentivirus-mediated delivery of galectin-3 shRNA ameliorated CIA in rats [75]. Downregulation of galectin-3 inhibited IL-6 secretion in FLSs from RA synovium [102].
**Galectin-9**	Galectin-9 deficiency promoted Th1 and Th17; and inhibited Treg differentiation, rendering susceptibility to CIA [103]. Galectin-9 induced apoptosis of FLS and downregulated pro-inflammatory cytokine production [104]. Galectin-9 ameliorated immune-complex-induced arthritis by regulating the expression profile of macrophage Fc receptors [118]	Galectin-9 induced apoptosis of FLS from RA patients in cell culture [104]. Decreased expression of galectin-9 was detected in RA patients with high disease activities [119]. Down-regulated expression of Tim-3 led to defective galectin-9-induced apoptosis of CD4^+^ T cells [120].	Administration of galectin-9 ameliorated CIA or immune complex-induced arthritis [104,118].
**Galectin-2**		Galectin-2 3279C/T gene polymorphism is correlated with diastolic blood pressure in patients with RA [123].	
**Galectin-8**		CD44vRA, a CD44 variant prevalent in RA patients, can neutralize the galectin-8 induced apoptosis of synoviocytes [125]. Autoantibodies against galectin-8 were detected in the sera of about 20% of RA patients [126,127]. A galectin-8 gene variant is prevalent in RA patients and associates with the early onset of RA [128]	
